# Identification of Cardiac CircRNAs in Mice With CVB3-Induced Myocarditis

**DOI:** 10.3389/fcell.2022.760509

**Published:** 2022-02-07

**Authors:** Xiang Nie, Jiahui Fan, Huihui Li, Jin Wang, Rong Xie, Chen Chen, Dao Wen Wang

**Affiliations:** Division of Cardiology, Hubei Key Laboratory of Genetics and Molecular Mechanisms of Cardiological Disorders, Tongji Hospital, Tongji Medical College, Huazhong University of Science and Technology, Wuhan, China

**Keywords:** fulminant myocarditis, CircRNAs, coxsackie B3 virus, inflammation, acute myocarditis

## Abstract

**Background:** Viral myocarditis could initiate various immune response to the myocardium, resulting in myocyte damage and subsequent cardiac dysfunction. The expression profile and functions of circRNAs in this process are unknown.

**Methods:** Fulminant myocarditis (FM) and non-FM models were induced by coxsackie B3 virus (CVB3) infection in A/J mice and C57BL/6 mice, respectively. CircRNAs expression profile was identified by RNA-seq. Quantitative RT-PCR, Spearman rank correlation, KEGG pathway, GO analysis, Western blot and flow cytometry were performed for functional analysis.

**Results:** Severer inflammatory cell infiltration and cardiomyocyte necrosis were presented in CVB3-treated A/J mice than those in C57BL/6 mice. The dysregulated circRNAs in both of the mouse strains displayed strong correlation with the immune response, but dysregulated circRNAs in A/J mice were more prone to cardiac dysfunction. KEGG analysis indicated that the target genes of dysregulated circRNAs in A/J mice were mainly involved in viral infection, T cell and B cell receptor signaling pathways, while the target genes of dysregulated circRNAs in C57BL/6 mice were unrelated to immune pathways. Furthermore, knockdown of circArhgap32 that was downregulated in CVB3-treated A/J mice promoted cardiomyocyte apoptosis *in vitro*.

**Conclusion:** Our data showed that cardiac circRNAs dysregulation is an important characteristic of viral myocarditis.

## Introduction

Acute myocarditis (AM) has been defined as a clinical manifestation of cardiac inflammation leading to myocardial damage, fibrosis and heart failure (Gupta et al., 2008). There are a variety of causes for AM, including viruses, bacteria, toxins and autoimmune diseases, among which enterovirus (especially coxsackie B3 virus, CVB3) was the most common pathogen (Maisch et al., 2014). Patients with myocarditis may present a wide range of symptoms, ranging from mild dyspnea or chest pain to cardiogenic shock and even death (Cooper, 2009). Infection by cardiotropic viruses triggers different symptoms, varying from severe myocarditis to mild or asymptomatic presentation. Fulminant myocarditis (FM), a severe form of myocarditis, has higher rates of cardiac death and heart transplantation compared with patients with non-FM (NFM) (Ammirati et al., 2019). Varying degrees of myocardial inflammation and cardiomyocyte necrosis are pathological features of AM (Ammirati et al., 2018). The molecular mechanisms underlying the various clinical presentation and severity of AM are largely elusive. It was well-recognized that A/J mice showed a higher susceptibility towards viral infection than C57BL/6 mice. CVB3 infected A/J mice exhibit severe myocarditis at day 3 and the viremia which persist up to 5–7 days after infection (Muller et al., 2015). Even after 21 days, severe heart lesions were still found in A/J mice (Wolfgram et al., 1986). Whereas the CVB3-resistant strains such as C57BL/6 mice could eliminate the virus after the early acute phase. Although histological examination showed regional myocardial inflammatory infiltrations in the initial stage, almost all signs of inflammation in the hearts of CVB3-treated C57BL/6 mice disappeared 21 days after infection (Turk, 1978; Gauntt and Huber, 2003; Corsten et al., 2012; Garmaroudi et al., 2015).

Recent studies have revealed that multiple non-coding RNAs participated in AM progression. Expression profiles of lncRNAs and miRNAs were identified in both children and adults with FM (Liu et al., 2019; Nie et al., 2020; Wang et al., 2021). A novel plasma miRNA hsa-miRChr8:96 could be used to distinguish patients with myocarditis from those with myocardial infarction, and the expression level of hsa-miRChr8:96 was positively correlated with the severity of myocarditis (Blanco-Domínguez et al., 2021). However, the involvement of circRNAs in AM pathogenesis remains largely unknown.

CircRNAs are a novel class of non-coding RNAs (ncRNAs), which originate from pre-mRNAs (Kristensen et al., 2019). CircRNAs are widely expressed in eukaryotic cells with high abundance, stable structure and tissue-specific pattern (Jeck and Sharpless, 2014). CircRNAs are emerging powerful regulators in cardiovascular diseases (Viereck and Thum, 2017; Huang et al., 2019), as well as in autoimmune diseases (Chen et al., 2019; Zhang et al., 2020a; Saaoud et al., 2020). For example, overexpression of circPPM1F could promote pancreatic islet injury by enhancing M1 macrophage activation through the circPPM1F-HuR-PPM1F-NF-κB axis (Zhang et al., 2020b). CircHIPK3 expression was significantly upregulated in myocardial tissue when exposed to LPS, while knockdown of circHIPK3 efficiently alleviated LPS-induced myocarditis in C57BL/6 mice (Fan et al., 2020). Nevertheless, expression patterns and functions of circRNAs in myocardial immune response and cardiac function remained elusive.

In the present study, circRNAs expression profiles were identified in A/J and C57BL/6 myocarditis mice models. CircRNAs showed diverging expression patterns, and might play important roles in the pathogenesis of viral myocarditis. Importantly, silencing of circArhgap32 that was downregulated in CVB3-induced A/J mice promoted cardiomyocytes apoptosis *in vitro*.

## Results

### Identification of Differentially Expressed CircRNAs in Viral FM and NFM

**FIGURE 1:**
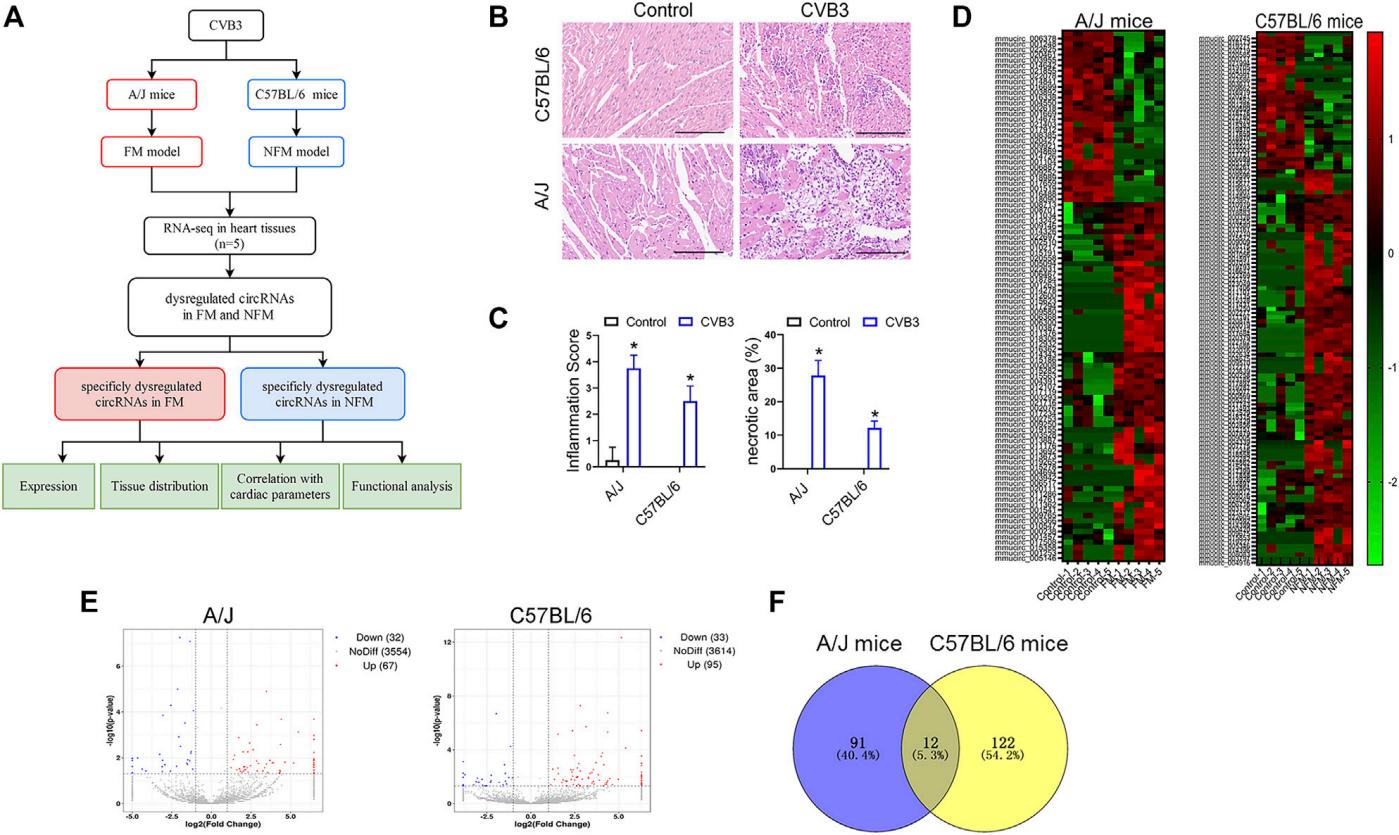
Identification of differentially expressed circRNAs in viral FM and NFM. **(A)** The flowchart of the present study. **(B)** Cardiac leukocyte infiltration and myocyte necrosis were detected by H&E staining in A/J or C57BL/6 mice respectively. **(C)** Inflammation score and necrosis area were performed for evaluating the severity of myocarditis; *p < 0.05. **(D)** Hierarchical cluster analysis of circRNAs between CVB3 and control groups. Red strip, high relative expression; green strip, low relative expression; Color brightness reflects the degree of expression increase or decrease. **(E)** Volcano plot of the expression of circRNAs in heart tissues. The red or blue plots represent up- or down-regulated circRNAs with *p* < 0.05, whereas the black plots represent insignificant changes. **(F)** Venn diagrams showed overlap of abnormally expressed circRNAs between A/J and C57BL/6 myocarditis models.

As reported previously, A/J mice are more susceptible to CVB3-induced myocarditis than C57BL/6 mice (Gauntt and Huber, 2003). To identify the role of circRNAs in viral myocarditis, we first analyzed the circRNA expression profile of CVB3-induced FM in A/J mice and NFM in C57BL/6 mice. The overall design of the present study was shown in Figure 1A. Severer inflammatory cell infiltration and cardiomyocyte necrosis were presented in CVB3-treated A/J mice than in C57BL/6 mice (Figures 1B,C).

The heat maps showed different expression patterns of circRNAs in the two mouse strains (Figure 1D). Totally, 67 circRNAs were upregulated and 32 circRNAs were downregulated in the hearts of CVB3-treated A/J mice, while 95 circRNAs were upregulated and 33 circRNAs were downregulated in the hearts of CVB3-treated C57BL/6 mice (Figure 1E). Venn diagrams showed only a small number of overlapped abnormally expressed circRNAs between the two mouse strains (Figure 1F).

The dysregulated circRNAs, which were specifically expressed in CVB3-treated A/J mice or C57BL/6 mice, might be associated with the diverging molecular response to CVB3 infection and the distinct severity in FM and NFM.

### Validation of Differentially Expressed CircRNAs in Viral FM and NFM

**FIGURE 2:**
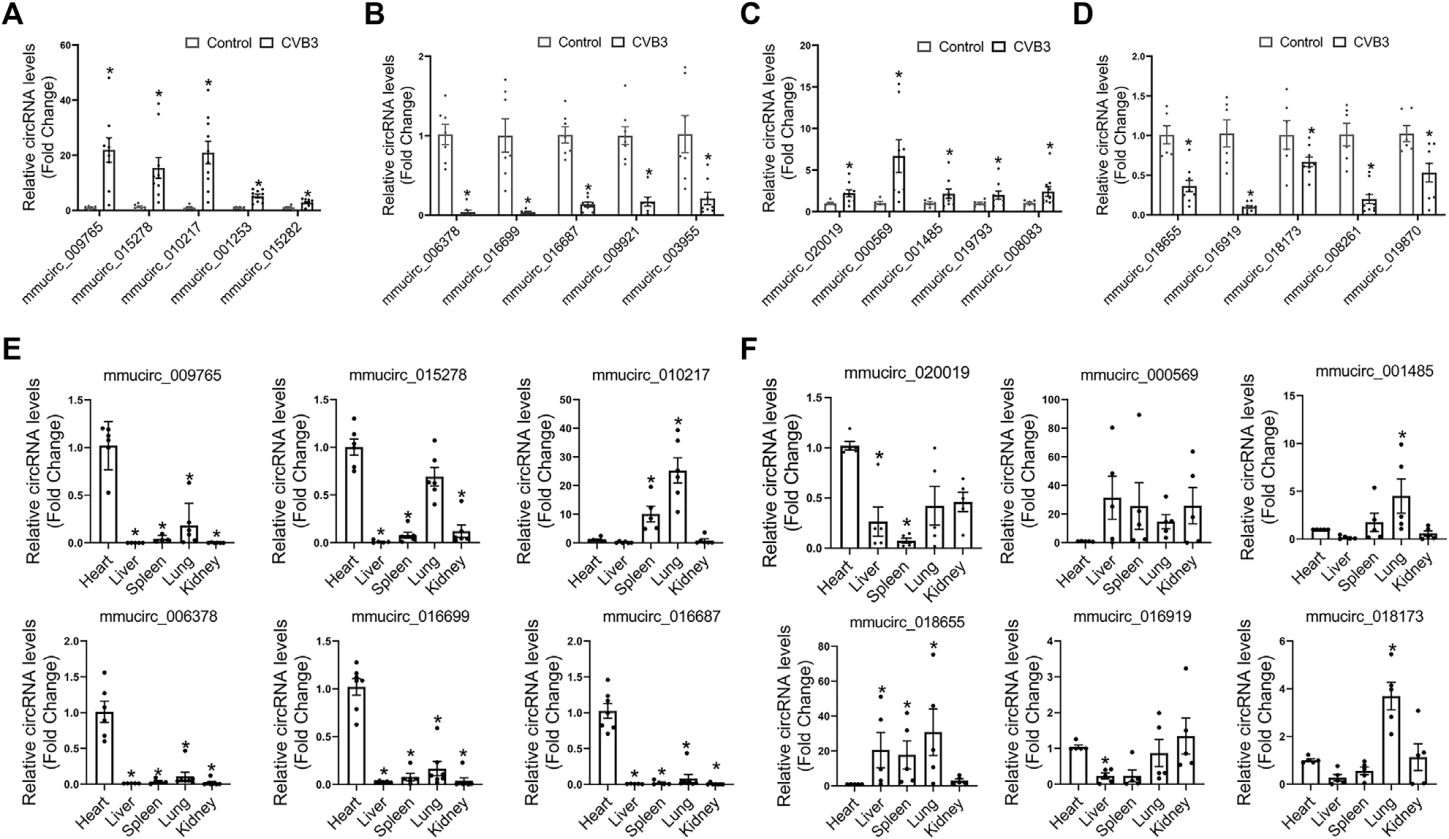
Validation of differentially expressed circRNAs in FM and NFM. **(A,B)** A/J-specific upregulated **(A)** and downregulated circRNAs **(B)** were verified by qRT-PCR in another heart samples; *p < 0.05 vs. Control. **(C,D)** C57BL/6-specific upregulated **(C)** and downregulated **(D)** circRNAs were verified by qRT-PCR in another heart samples; *p < 0.05 vs. Control. **(E,F)** The tissue distribution of A/J-specific **(E)** and C57BL/6-specific **(F)** dysregulated circRNAs; *p < 0.05 vs. Heart.

To validate the differentially expressed circRNAs and explore their functions in FM and NFM, we firstly performed qRT-PCR assays to measure the expression levels of the top five dysregulated circRNAs in either CVB3-treated A/J mice or CVB3-treated C57BL/6 mice, respectively. Consistently, mmucirc_009765, mmucirc_015278, mmucirc_010217, mmucirc_001253, and mmucirc_015282 were increased, while mmucirc_006378, mmucirc_016699, mmucirc_016687, mmucirc_009921, and mmucirc_003955 were decreased in CVB3-treated A/J mice (Figures 2A,B). Meanwhile, up-regulation of mmucirc_020019, mmucirc_000569, mmucirc_001485, mmucirc_019793, and mmucirc_008083, as well as down-regulation of mmucirc_018655, mmucirc_016919, mmucirc_018173, mmucirc_008261, and mmucirc_019870 were observed in CVB3-treated C57BL/6 mice (Figures 2C,D). Moreover, the expression levels of most of these circRNAs were also dysregulated in cultured cardiomyocytes upon CVB3 infection which were in line with the RNA-seq data in animal models, suggesting that these circRNAs might be cardiomyocyte-derived (Supplementary Figure S1A).

Further, we evaluated the tissue distribution of these dysregulated circRNAs in the two mouse strains without any stress. The dysregulated circRNAs in CVB3-treated A/J mice, including mmucirc_009765, mmucirc_015278, mmucirc_006378, mmucirc_016699, and mmucirc_016687, were mainly enriched in the heart tissue as compared to other organs (Figure 2E). However, the dysregulated circRNAs in CVB3-treated C57BL/6 mice were widely expressed in all organs tested, among which mmucirc_000569, mmucirc_001485, and mmucirc_018655 showed little abundance in the heart (Figure 2F).

These results suggested that circRNA dysregulation and divergent tissue distribution were important features of AM, which might determine the severity of cardiac inflammation and function.

### Correlation Between CircRNAs Expression Levels and Cardiac Inflammation

**FIGURE 3:**
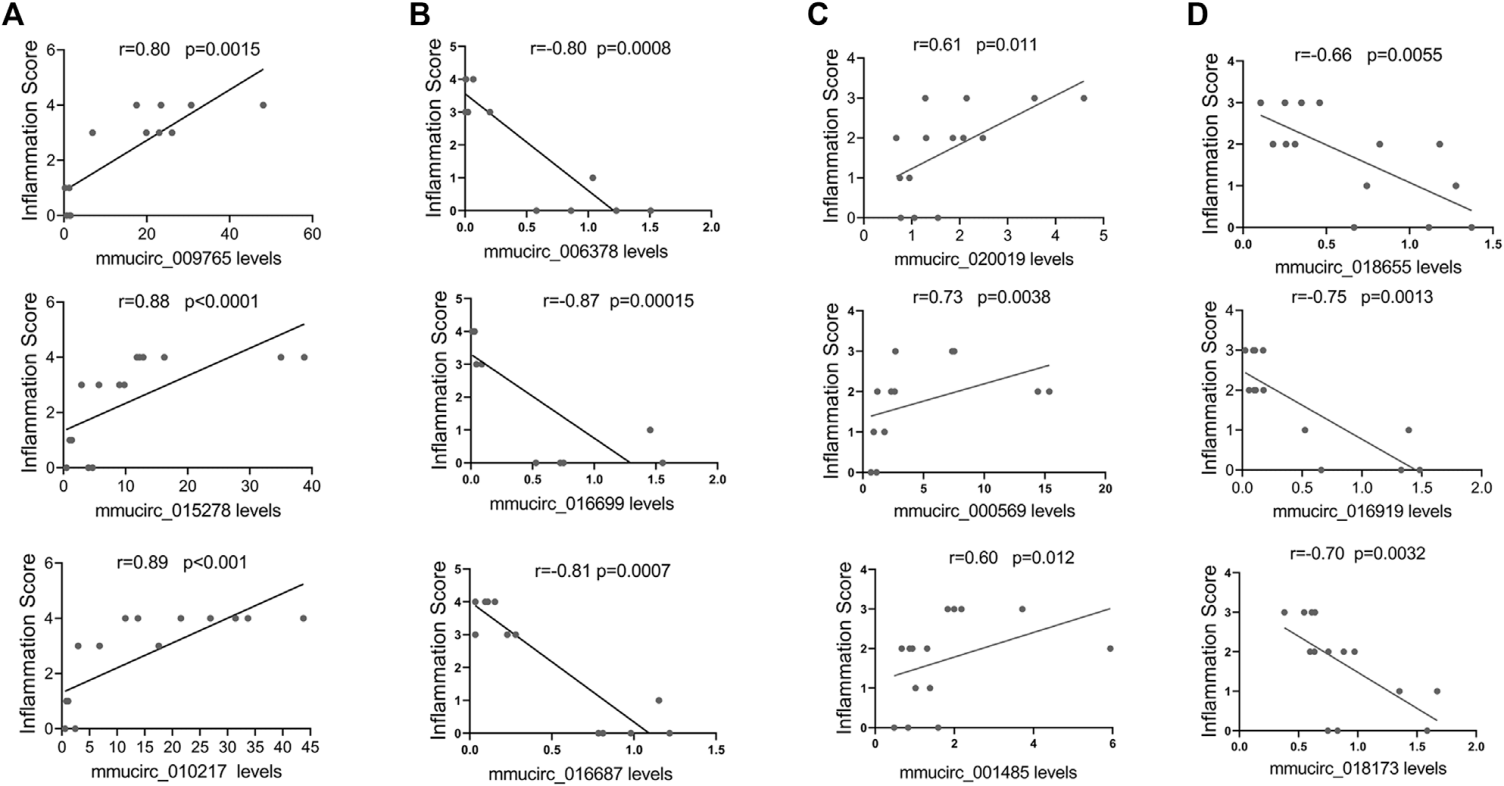
Correlation between circRNAs expression levels and cardiac inflammation. **(A)** The correlation between A/J-specific upregulated circRNAs (mmucirc_009765, mmucirc_015278, mmucirc_010217) and inflammatory response, N > 12. **(B)** The correlation between A/J-specific downregulated circRNAs (mmucirc_006378, mmucirc_016699, mmucirc_016687) and inflammatory response, N > 12. **(C)** The correlation between C57BL/6-specific upregulated circRNAs (mmucirc_020019, mmucirc_000569, mmucirc_001485) and inflammatory response, N > 12. **(D)** The correlation between C57BL/6-specific downregulated circRNAs (mmucirc_018655, mmucirc_016919, mmucirc_018173) and inflammatory response, N > 12.

Then, linear correlation analysis was performed between circRNAs levels and inflammatory infiltration to evaluate the potential roles of circRNAs in cardiac immune response. In CVB3-treated A/J mice, the top three upregulated circRNAs, such as mmucirc_009765, mmucirc_015278, mmucirc_010217, displayed strong positive correlation with cardiac immune response; while the top three downregulated circRNAs, such as mmucirc_006378, mmucirc_016699, mmucirc_016687 were negatively correlated with cardiac immune response (Figures 3A,B). On the other hand, in CVB3-treated C57BL/6 mice, the top six dysregulated circRNAs, including mmucirc_020019, mmucirc_000569, mmucirc_001485, mmucirc_018655, mmucirc_016919, mmucirc_018173 showed mild correlation with cardiac immune response (Figures 3C,D).

These data indicated that circRNAs might play a vital role in cardiac immune response in both FM and NFM.

### Correlation Between CircRNAs Expressions and Cardiac Function

**FIGURE 4:**
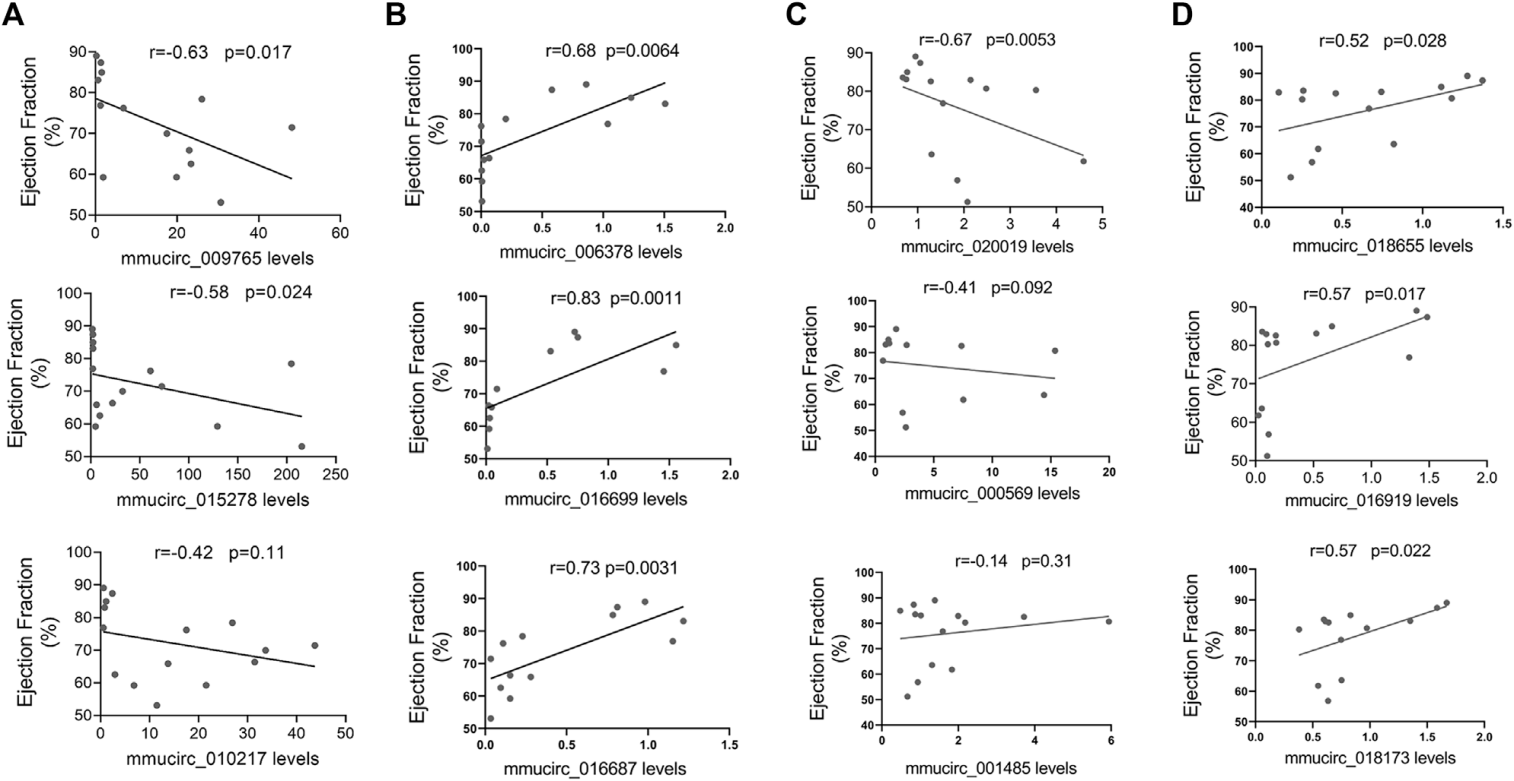
Correlation between circRNAs expression levels and cardiac function. **(A)** The correlation between A/J-specific upregulated circRNAs (mmucirc_009765, mmucirc_015278, mmucirc_010217) and cardiac function, N > 12. **(B)** The correlation between A/J-specific downregulated circRNAs (mmucirc_006378, mmucirc_016699, mmucirc_016687) and cardiac function, N > 12. **(C)** The correlation between C57BL/6-specific upregulated circRNAs (mmucirc_020019, mmucirc_000569, mmucirc_001485) and cardiac function, N > 12. **(D)** The correlation between C57BL/6-specific downregulated circRNAs (mmucirc_018655, mmucirc_016919, mmucirc_018173) and cardiac function, N > 12.

Moreover, the possibility that these selected circRNAs affect cardiac function was also explored. In line with the heart-enriched expression pattern, the expression of five specific dysregulated circRNAs in CVB3-treated A/J mice, including mmucirc_009765, mmucirc_015278, mmucirc_006378, mmucirc_016699, and mmucirc_016687, illustrated a strong correlation with left ventricular ejection fraction (Figures 4A,B). While in CVB3-treated C57BL/6 mice, only four dysregulated circRNAs, namely mmucirc_002219, mmucirc_0018655, mmucirc_016919, and mmucirc_0018173, were correlated with left ventricular ejection fraction (Figures 4C,D).

In general, these dysregulated circRNAs, especially the dysregulated circRNAs in CVB3-treated A/J mice, might participate in the regulation of cardiac immune response.

### Bioinformatic Analysis of Dysregulated CircRNAs in Viral Myocarditis

**FIGURE 5:**
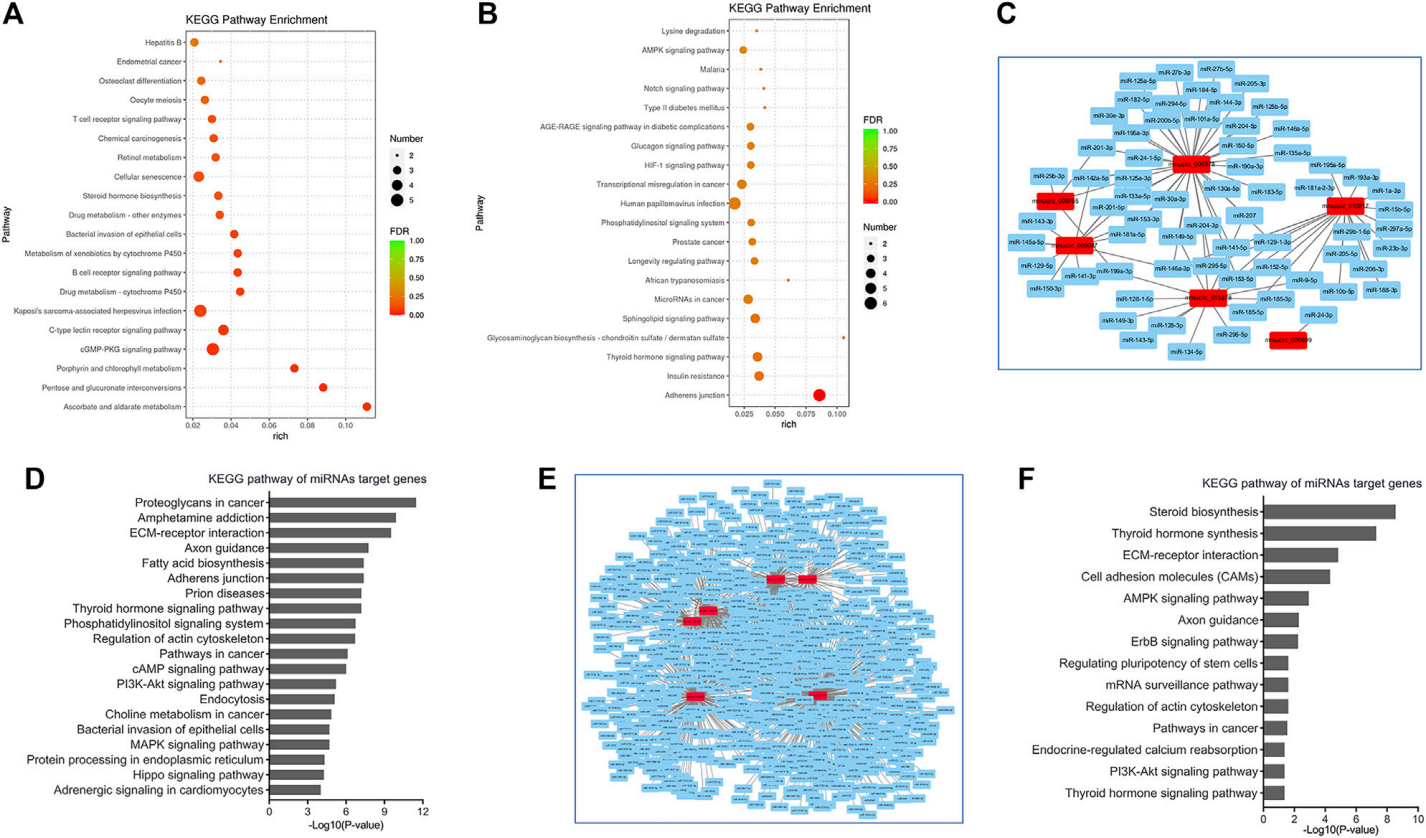
Bioinformatic analysis of circRNAs in viral myocarditis. **(A,B)** Kyoto Encyclopedia of Genes and Genomes (KEGG) pathway analyses for cis-target genes of A/J-specific **(A)** and C57BL/6-specific **(B)** dysregulated circRNAs. **(C)** CircRNA-miRNAs networks for A/J-specific dysregulated circRNAs. **(D)** KEGG pathway analyses for miRNAs targeted by A/J-specific dysregulated circRNAs. **(E)** CircRNA-miRNAs networks for C57BL/6-specific dysregulated circRNAs. **(F)** KEGG pathway analyses for miRNAs targeted by C57BL/6-specific dysregulated circRNAs.

Functional analysis of differentially expressed circRNAs was undertaken by GO and KEGG pathway enrichment based on the differentially expressed source gene of circRNAs to identify the cis-regulated genes. KEGG pathway analysis indicated that the target genes of these A/J-specific circRNAs were mainly involved in immune pathways, such as viral infection (Hepatitis B and Kaposi’s sarcoma−associated herpesvirus), T cell and B cell receptor signaling pathways; while the target genes of the C57BL/6-specific dysregulated circRNAs were involved in human papillomavirus infection, adherens junction and AMPK signaling pathways but absent in immune related pathways (Figures 5A,B). GO analysis of all these dysregulated circRNAs showed that pathways such as innate immune response or response to virus were activated in CVB3-induced A/J mice but not in C57BL/6 mice (Supplementary Figure S1B).

Moreover, circRNAs could also sponge miRNAs to exert biological functions. mirPath analyses revealed that inflammation-related or cardiac injury-associated miRNAs such as miR-129 (Meng et al., 2020), miR-1 (Yang et al., 2007), miR-150 (Zhu et al., 2020), etc. were also enriched in A/J-specific circRNAs. Target genes of these miRNAs were mainly involved in PI3K-Akt signaling pathway, MAPK signaling pathway, Hippo signaling pathway, and Adrenergic signaling in cardiomyocytes (Figures 5C,D).

Although a number of miRNAs, such as miR-6539, miR-6900-3p, miR-7003-3p and miR-883a-5p, were identified as targets of C57BL/6-specific circRNAs, their functions are rarely studied. These miRNAs were mainly involved in steroid biosynthesis, thyroid hormone synthesis, AMPK signaling pathway, and PI3K-Akt signaling pathway (Figures 5E,F).

Together, the target genes of A/J-specific circRNAs were more enriched in the genes involved in immune response and cardiac function.

### Silencing of CircArhgap32 Promotes Cardiomyocytes Apoptosis

We found that A/J-specific dysregulated circRNAs might play a more important role in regulating cardiac function during AM, among which mmucirc_016699 showed the strongest correlation with cardiac function, and were mainly expressed in the heart. Besides, the expression level of circArhgap32 was mainly reduced in cardiomyocytes upon CVB3 infection (Supplementary Figure S1C). To validate the predicted potential biological function of these circRNAs, we focused on mmucirc_016699, which was derived from the arhgap32 gene (Rho GTPase activating protein 32) and termed as circArhgap32.

**FIGURE 6:**
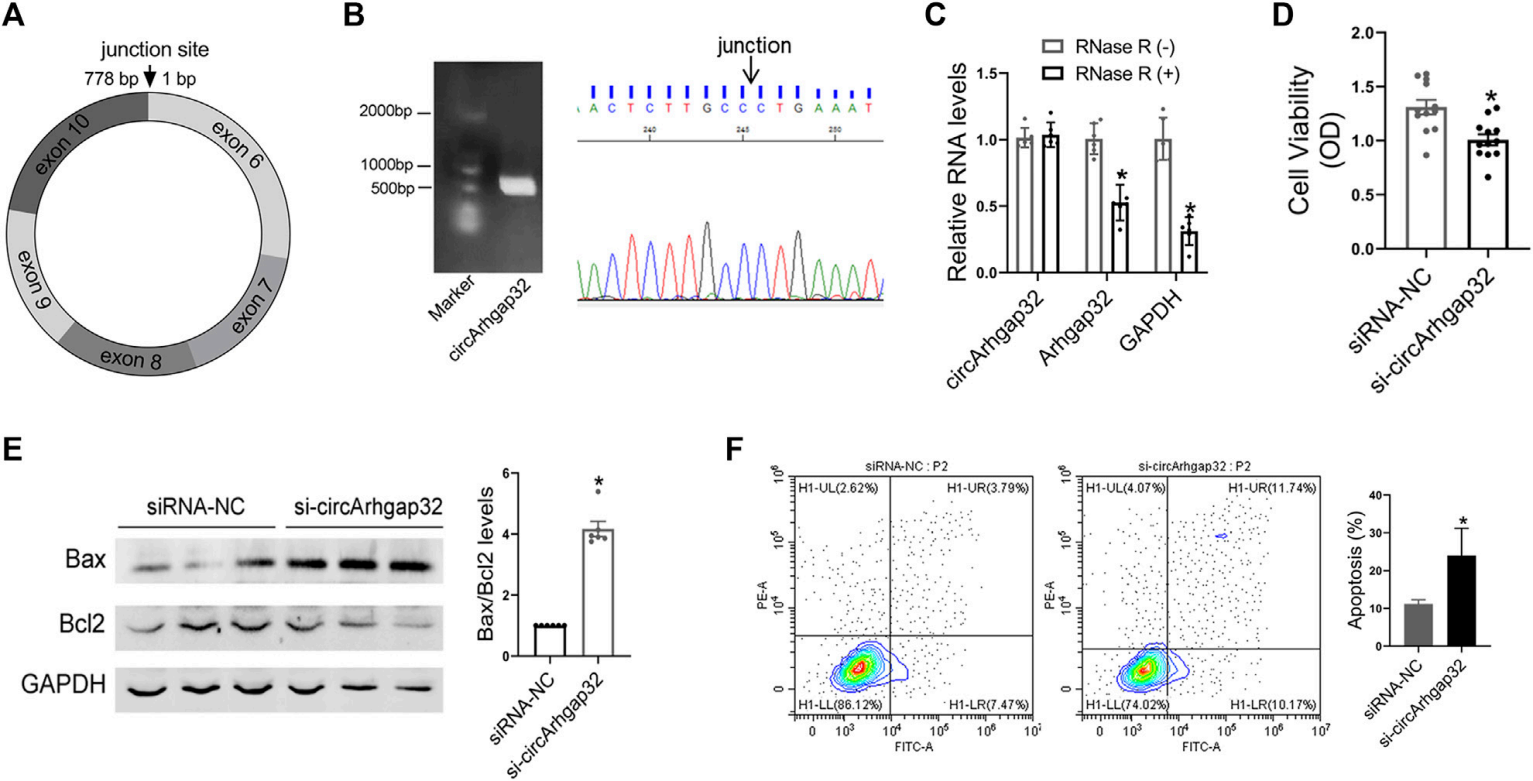
Silencing of circArhgap32 promotes cardiomyocytes apoptosis. **(A)** Schematic illustrating the structure of circSnx5. **(B)** CircArhgap32 transcript was validated by Sanger sequencing. **(C)** Total RNA was digested with or without RNase R, followed by qRT-PCR measurements of circArhgap32, Arhgap32 and GAPDH; *p < 0.05 vs. RNase R (−). **(D)** Cell viability was detected in si-Arhgap32 or siRNA-NC treated HL-1 cells; *p < 0.05 vs. siRNA-NC. **(E)** Western blot for Bax and Bcl2 expression in si-Arhgap32 or siRNA-NC treated HL-1 cells; *p < 0.05 vs. siRNA-NC. **(F)** Cell apoptosis was detected by flow cytometry; *p < 0.05 vs. siRNA-NC.

According to circBase database annotation, circArhgap32 was spliced from arhgap32 gene on chr9:31959506-32015850 and had a final-form length of 778 nt (Figure 6A). Divergent primers amplified circArhgap32 in complementary DNA but not genomic DNA, indicating that this RNA was in circular form (Figure 6B). Furthermore, in RNase R digestion experiments, the circular nature of circArhgap32 was confirmed by showing resistance to RNase R digestion (Figure 6C).

In order to determine the effects of circArhgap32 on cardiomyocyte, knocking down endogenous circArhgap32 by small interfering RNAs (siRNAs) that specifically target the circular junction of circArhgap32 was performed. The qRT-PCR analysis indicated that circArhgap32 siRNA (si-circArhgap32) transfection significantly downregulated endogenous circArhgap32 level, without affecting its linear counterparts (Arhgap32 gene) (Supplementary Figure S1D). circArhgap32 silencing could promote proinflammatory cytokine production, such as IL-1β and TNF-α (Supplementary Figure S1E). Furthermore, circArhgap32 downregulation impaired cardiomyocyte’s viability and promoted cell apoptosis, indicating that circArhgap32 plays a vital role in cardiac function (Figures 6D–F).

## Discussion

The current study compared the circRNA expression profile related to immune response and cardiac function between CVB3-induced FM in A/J mice and NFM in C57BL/6 mice, respectively. The results indicated that circRNA dysregulation is an important characteristic of AM.

The initial pathogenesis of both FM and NFM is considered to be similar, regardless of the triggers including viral infections, toxic substances and autoimmune conditions. FM is the most severe type of myocarditis and is characterized by acute onset and rapid progress. FM might present with abnormity in hemodynamics, severe heart failure, or even death (Sharma et al., 2019). In contrast to patients with NFM who present with New York Heart Association (NYHA) class II-III symptoms of heart failure, patients with the FM exhibit symptoms of heart failure that meet NYHA class IV criteria (Cooper, 2009). Patients with FM exhibit a greater inflammatory involvement of the myocardium and a greater extent of edema and fibrosis as well as more severely impaired LVEF than those with NFM (Ammirati et al., 2019). Cardiomyocyte death and extensive inflammation are the predominant histopathological changes during FM (Ryu et al., 2013). Biomarkers of cardiac injury are elevated in patients with acute myocarditis. A number of biomarkers can assist in diagnosing FM and estimating extent of disease. FM have higher plasma concentrations of C-reactive protein and Creatinine kinase MB than NFM (Smith et al., 1997). FM patients should receive active symptomatic management and life-support treatment (Wang et al., 2019). FM is associated with overall worse outcomes that include lower LVEF at last follow-up, higher in-hospital mortality, and increased rates of cardiac transplantation (Sagar et al., 2012).

Viral infection is the most common cause of myocarditis. CVB3, a cardiotropic virus, is the most common etiology for myocarditis (Garmaroudi et al., 2015); (). CVB3-induced acute myocarditis includes early myocyte damage. CVB3 infection begins by coupling of the virus to host-cell receptors such as CAR (coxsackievirus, and adenovirus receptor), and DAF (decay-accelerating factor). CVB3 variant PD can replicate in DAF and CAR negative cells suggesting that other receptors such as heparan sulfate are also involved in attachment of virus to the host cells (Rivadeneyra et al., 2018). Once entering into host cells, viral RNA was rapidly translated and the virus was packaged, followed by lysis of host cells, mainly through apoptosis, necrosis and autophagy. Besides the direct injury effect, aberrant immune response-mediated host cell damage was also involved in CVB3 infection. CVB3 might be sensed by well-known pattern recognition receptors (PPRs) including the Toll-like receptors (TLRs) and the RIG-I-like RNA helicases (RLHs), especially TLR3, TLR4 and MDA5 (Massilamany et al., 2014; Weithauser et al., 2016). Following ligand recognition, these receptors initiate an intracellular signaling cascade resulting in the production of type-1 IFN and pro-inflammatory cytokines such as TNF-α, IL-1, IL-18 and IL-4. Signal-transduction pathways such as GSK3β/β-catenin, MAPKs/ERK, NF-κB signal pathway and survival pathways such as protein kinase B or Akt were also significantly activated upon CVB3 infection in host cells (Daba et al., 2019).

Over-activated immune responses evoked by viral infection as well as subsequent cardiac myocyte destruction, reparative fibrosis, and heart failure are important for the pathogenesis of myocarditis, especially in FM (Fung et al., 2016). Acute myocarditis with multi-organ dysfunction is more common in young children, whereas adult patients usually present with asymptomatic, with only a few presenting with acute myocarditis (Sagar et al., 2012). The annual incidence of acute myocarditis is estimated at approximately 22 cases per 100,000 population, with heart failure just 4% of these cases (Vos et al., 2015). In studies of patients hospitalized with myocarditis, approximately 30% were considered as fulminant myocarditis (Ammirati et al., 2017). Much of our understanding of the pathophysiology and mechanisms of human viral myocarditis is based on studies of experimental murine models. Susceptible mouse strains (A/J; BALB/c) develop severe myocarditis as represented by the sustained presence of viral RNA within the myocardium, whereas more resistant strains such as C57BL/6 are able to eliminate the virus after the early acute phase (Gauntt and Huber, 2003; Corsten et al., 2012; Garmaroudi et al., 2015). The different gene backgrounds of mice showed variable susceptibility for viral infection. CVB3-treated A/J mice usually exhibit severer inflammatory infiltration and cardiac dysfunction even at 10^4^ PFU virus, which is considered as a model of severe myocarditis, while C57BL/6 mice was recognized with hereditary low susceptibility to virus-induced myocarditis at 10^5^ PFU (Althof et al., 2018). A/J mice with virus at 10^4^ PFU alone showed high mortality over time (Nakamura et al., 2013). Low dose of virus at 5.0 × 10^1^ TCID_50_ resulted in 20% mortality of the infected A/J mice, whereas doses ranging from 1 × 10^3^ to 1 × 10^5^ TCID_50_ led to high mortalities (89–100%) as early as 5–14 days, and the inflammatory foci was similar at 1.0 × 10^3^ and 1.0 × 10^5^ TCID_50_ (Gangaplara et al., 2012). CVB3 infection at 10^4^ PFU in C57BL/6 mice displayed mild inflammatory infiltration and less than 20% mortality (De Giusti et al., 2015; Huang et al., 2018). What determines the variable clinical presentation and severity of this disease are largely unknown.

Activation of inflammatory cells and immune response is important to eliminate viral infection, but over-activation of inflammatory reaction may also lead to adverse inflammatory cytokines secretion and result in severe myocardial damage. Viral entry into the target cells is facilitated by host receptors such as decay-accelerating factor and coxsackievirus-adenovirus receptor, which cause myocardial injury *via* apoptosis and necrosis of cardiomyocytes within 3–4 days post-infection (Garmaroudi et al., 2015; Rivadeneyra et al., 2018). Upon infection, various cardiac-resident cells, such as cardiomyocytes, endothelial cells, mast cells and fibroblasts, may contribute to acute inflammation by secreting cytokines such as IL-1, IL-6, TNF-α and IL-18 (Tschöpe et al., 2021). Innate leukocytes may produce type I IFNs to prevent viral replication after infection. Over-activation of NK cells may exacerbate myocardial damage owing to excessive release of cytotoxic molecules within the myocardium (Ong et al., 2017). The involvement of T cell subsets differs among various strains of mice and appears to be an important determinant of host susceptibility to viral myocarditis. Researchers showed that host susceptibility was correlated with the increased T cell responses, particularly those expressing the γδ T cell receptor (Huber et al., 1999; Esfandiarei and McManus, 2008). Th1 response is considered to be protective in acute myocarditis because it prevents viral replication (Uitendaal et al., 1979; Zhang et al., 2016). Th2 responses can reduce acute myocarditis by promoting T regulatory (Treg) cells and secreting IL-4 and IL-10 (Cunningham, 2001). Antibodies produced by B cells help to neutralize and clear the infectious virus within 2 weeks post-infection (Krebs et al., 2007). IL-10-producing B cells downregulate the proportion of Th1 and Th17 cells to alleviate inflammatory damage in the myocardium during AM (Wei et al., 2019). Hereditary susceptibility for viral infection, immune cells over-activation, and excessive release of cytotoxic molecules may be significant contributors in diverse pathogenesis of myocarditis.

Non-coding RNA (ncRNA) refers to RNA that is not translated into protein and plays a vital role in many biological processes. There is expanding evidence revealing that ncRNAs regulate the occurrence and development of AM (Liu and Ding, 2017; Zhang et al., 2020a). Dysregulated miRNA expressions were detected in the plasma from FM patients, and miR4763-3p could serve as a potential biomarker for FM diagnosis (Nie et al., 2020). MiR-21 and miR-146b were upregulated in a mouse model of AM, inhibition of miR-21 and miR-146b decreased the expression levels of IL-17 and RORγt (Liu et al., 2013). Expression profile of lncRNAs in the heart of mice with coxsackie B3 virus-induced myocarditis was also explored, and lncRNA expression levels displayed a strong correlation with immune response and cardiac function. LncRNA AK085865 promoted macrophage M2 polarization in CVB3-induced viral myocarditis by regulating ILF2-ILF3 signaling pathway (Zhang et al., 2020c). CircRNAs are considered as critical modulators in immune and inflammatory reactions (Zhang et al., 2020a). CircKcnt2 recruits the nucleosome remodeling deacetylase (NuRD) complex onto Batf (basic leucine zipper transcription factor) promoter to suppress its expression, which inhibits ILC3 activation to promote innate colitis resolution (Liu et al., 2020). Overexpression of circPPM1F could promote pancreatic islet injury by enhancing M1 macrophage activation through the HuR-PPM1F-NF-κB axis (Zhang et al., 2020b). CircSirt1 inhibits vascular inflammation by sequestering NF-κB p65 in the cytoplasm (Yang et al., 2007). Circ_001253, termed as circ_SIPA1L1, was aberrantly elevated in osteosarcoma tissues and cell lines, and over-expression of circ_SIPA1L1 promotes osteosarcoma progression *via* miR-379-5p/MAP3K9 axis (Jun et al., 2020). Circ-RHOT1 was increased in non-small cell lung cancer. Circ-RHOT1 overexpression abolished proliferation, migration, and invasion induced by propofol in non-small cell lung cancer by regulating miR-326 (Huang et al., 2020; Zhang et al., 2021).

Here, we reported the expression patterns of circRNAs in heart tissues during AM. A set of circRNAs were dysregulated in mild and severe myocarditis respectively. In A/J mice, a severe form of myocarditis, specifically dysregulated circRNAs were strongly correlated with the immune response and cardiac function during AM. Functional analyses showed that immune pathways, such as viral infection and T cell or B cell receptor signaling pathways, were notably activated in A/J-specific dysregulated circRNAs. Silencing of circArhgap32 impaired cardiomyocyte viability and promoted cell apoptosis. These results indicated that circRNAs dysregulation was an important characteristic of AM and may determine the severity of myocarditis. However, there are still limitations in this study. It should be mentioned that results might vary across the genetic backgrounds, environmental factors, platforms and sample sizes. Further in-depth studies are ongoing to demonstrate the exact biological functions of circRNAs in different animal models of myocarditis.

Overall, this study provided a comprehensive expression profile of differentially expressed circRNAs in AM, whose expression levels were related to the severity of myocarditis, and functional analyses indicated potential mechanisms of dysregulated circRNAs in AM.

## Materials and Methods

### Animal Study

All animal studies were in accordance with the NIH Guide for the Care and Use of Laboratory Animals and conducted with the approval of the Animal Research Committee of Tongji Medical College. Male A/J mice (∼6-week-old) were purchased from GemPharmatech (Nanjing, China). Male C57BL/6 (∼6-week-old) mice were purchased from Beijing Vital River Laboratory Animal Technology (Beijing, China). A/J and C57BL/6 mice were randomly assigned into two groups: Controls (*n* = 6) and CVB3-treated groups (*n* = 10), respectively. C57BL/6 mice were injected with 10^5^ PFU CVB3, while A/J mice were injected with 10^4^ PFU CVB3 intraperitoneally. Control groups were injected with PBS alone.

### Cell Culture

HL-1 cells were cultured in Dulbecco’s modified Eagle’s medium (DMEM) supplemented with 10% fetal bovine serum (FBS) in a humidified atmosphere of 95% air and 5% CO_2_ at 37 °C. Cells were transfected with siRNA (RiboBio, China) targeting circArgap32 using Lipofectamine 6,000 (Beyotime, China) following the manufacturer’s protocol and collected 24 h later. Each experiment was repeated at least three times independently.

### RNA Isolation

All the mice were sacrificed on day 7 after CVB3 infection. Heart tissue samples were collected from A/J or C57BL/6 mice. Trizol Reagent (Invitrogen Life Technologies, CA) was used to extract total RNA and the concentration, quality, and integrity of RNAs were detected by NanoDrop spectrophotometer (Thermo Scientific, CA). Approximate 3 mg of RNA was used to generate RNA-Seq cDNA libraries using the TruSeq RNA Sample Preparation Kit (Illumina, San Diego, CA).

### CircRNA Sequencing Analysis

The sequencing was performed by Personal Biotechnology Co. (Shanghai, China) on a Hiseq X ten platform/NovaSeq 6,000 (Illumina, San Diego, CA). Cutadapt (v2.7) software was used to filter the sequencing data and obtain clean reads for further analysis. Reference genome index was built by Bowtie2 (v2.4.1) and high-quality sequences were mapped to the reference genome using HISAT2 (v2.1.0). The resulting *p*-values were adjusted using the Benjamini and Hochberg’s approach for controlling the false discovery rate. **CircRNAs** with an adjusted *p* < 0.05 and absolute logFC >1 found by DESeq2 were assigned as differentially expressed. The datasets presented in this study can be found in online repositories. The names of the repository/repositories and accession number(s) can be found below: [the ArrayExpress database at EMBL-EBI (www.ebi.ac.uk/arrayexpress) under accession number E-MTAB-10823].

### Validation by Quantitative Real-Time PCR

Total RNA from mice heart tissue was transcribed into cDNA, and circRNAs expression was quantified by quantitative real-time PCR using Hieff qPCR SYBR Green Master Mix (Yeasen Biotech, China) on a 7900HT FAST real-time PCR system (Life Technologies, Carlsbad, CA). Divergent circRNAs primers were synthesized by AuGCT (Wuhan, China) (Supplementary Table S1). Relative expression levels were calculated with the 2^−ΔΔCt^ relative quantification method as previously described (Nie et al., 2020).

### Cardiac Function Detection in Mice

Cardiac function was detected by echocardiography on day 7 after CVB3 infection using a high-resolution imaging system with a 30-MHz high-frequency scanhead (VisualSonics Vevo770, VisualSonics, Toronto, Canada) as described previously (Nie et al., 2018).

### Histological Analysis

The morphology of heart tissues was detected by H&E staining and measured by Image-Pro Plus Version 6.0 software (Media Cybermetics, Washington) as described previously (Fan et al., 2018). The inflammatory scores were defined as follows: 0, no inflammatory infiltrates; 1, small foci of inflammatory cells between myocytes; 2, larger foci of inflammatory cells; 3, >10% of a cross-section involved; 4, >30% of a cross-section involved (Mirna et al., 2021).

### Functional Analysis of Target Genes of Differently Expressed circRNAs

Cis-regulated source gene of circRNAs were predicted using the UCSC database. DAVID Bioinformatics Resources was used for GO and KEGG pathway analysis of the target genes. Statistical significance was set at *p* < 0.05. MiRNAs targets of circRNAs were predicted by miRnada and psRobot. Cytargetlinker was used in Cytoscape for the miRNA-mRNA network as described previously (Nie et al., 2021).

### Western Blotting Analysis

Western blotting was performed using the specific antibodies as described previously (Nie et al., 2018). The intensities of individual bands were analyzed by densitometry using ImageJ (National Institutes of Health software).

### Statistical Analysis

Data are shown as means ± SEM. Each data set was tested for normality typically using the Shapiro-Wilk test. Statistical analyses were then performed with Two tailed student’s t-test (parametric unpaired, two group of analysis), Mann-Whitney U test (non-parametric unpaired, two group of analysis). Kruskal–Wallis ANOVA test with Dunn’s multiple comparisons test (comparisons among groups more than two, non-parametric unpaired). Spearman rank correlation was conducted to evaluate the relationships between candidate circRNAs and cardiac parameters. Statistical tests were performed using GraphPad Prism (v8.0) (GraphPad Software, San Diego, CA) with *p* value <0.05 considered significant.

## Data Availability

The datasets presented in this study can be found in online repositories. The names of the repository/repositories and accession number(s) can be found below: ArrayExpress database at EMBL-EBI (www.ebi.ac.uk/arrayexpress) under accession number E-MTAB-10823.
