# Supplementary material for: Identification of Cardiac CircRNAs in Mice With CVB3-Induced Myocarditis
Source: Front Cell Dev Biol. 2022 Feb 7;10:760509. doi: 10.3389/fcell.2022.760509 (PMC8859109; doi:10.3389/fcell.2022.760509)
Supplement: Supplementary file 1 [file Table1.docx]

**Supplemental Table.**

| **Supplemental Table 1. The primers for circRNAs detection in mice.** | | |
| --- | --- | --- |
| **CircRNAs ID** | **Forward Primer (5’-3’)** | **Reverse Primer (5’-3’)** |
| mmucirc_009765 | CGACAGCCCGATACTGGAAG | AGATGGACAGACAGGCAAAA |
| mmucirc_015278 | TGAACCTGGAAGGGCTGGTA | ACTGCCATTCAGGGAATCCT |
| mmucirc_010217 | GGTCAATTTGAATGCATCTGTG | AGAAATATGACTTTGGTGGAGT |
| mmucirc_001253 | ACGTGGATCTTGGCGCATAC | CTTGCATCACTGGGGCTCTG |
| mmucirc_015282 | TAGAGCTGTGCCTCATTCAG | AATTGGCTAGTCATGGGTCT |
| mmucirc_006378 | GGGTGCTGGGATTAAAGGTG | TAAAGTGGCATAGAAGAAGTCTGCT |
| mmucirc_016699 | TTACCTGCCCTGCTGATTCCT | TCCAACTCTTGCCCTGGTTTT |
| mmucirc_016687 | GGAATACGTCACAGAACCCAAGA | GGCTTTAAGTCAAAGAAGGGAG |
| mmucirc_009921 | AAACCTAATGAGGCAGAAGAAAATT | CCCAAATACTCCAGGCAACG |
| mmucirc_020019 | ATGCCAACATCGACCTCACAA | AGAATGGAAGTATTTCAGCGTCTC |
| mmucirc_003955 | AGTGGTTCTACTTTGAAGTCAGTGG | CACTTTTAGCTTGCTCATCTTGAGT |
| mmucirc_000569 | TCCAGTCCTAACTTCAGTTGT | GATGGCTTCTGTTTTCATTAAATAA |
| mmucirc_001485 | ACTTCAACATTGTAATTCCGGATG | ATCAAAGGTGAATTCATGGGAGTAG |
| mmucirc_019793 | CAAATAGGAAATATCATTCGGGTTT | TAGGAGGTGTCCTAGCCTTAATCTT |
| mmucirc_008083 | GCCACAAAGTTGTAAGGGATAT | GCCCTCATTTTAGTTAGCTTGA |
| mmucirc_018655 | AACCTTCCTAAGGAGGCTAAAT | ATCATCATCCAACTTCATCACC |
| mmucirc_016919 | TCAAGCTCAAGAACGCCTAT | TGCTCAGTCAGGTCTGAGATC |
| mmucirc_018173 | ATCATGACTTTCCGCCCCACC | TGGGCACCCTGCGACTCAA |
| mmucirc_008261 | CGTTTGCAGCAGCCTTGGAC | CTCGCTTGCAGACTTCCTCACTT |
| mmucirc_019870 | TTAATTAAGCCTTTTTGATTGGTTG | CCAACATAACTCCAACATCATCAAC |
