# Supplementary material for: Identification of Cardiac CircRNAs in Mice With CVB3-Induced Myocarditis
Source: Front Cell Dev Biol. 2022 Feb 7;10:760509. doi: 10.3389/fcell.2022.760509 (PMC8859109; doi:10.3389/fcell.2022.760509)
Supplement: Supplementary file 2 [file DataSheet1.docx]

**Supplemental Figure 1.**


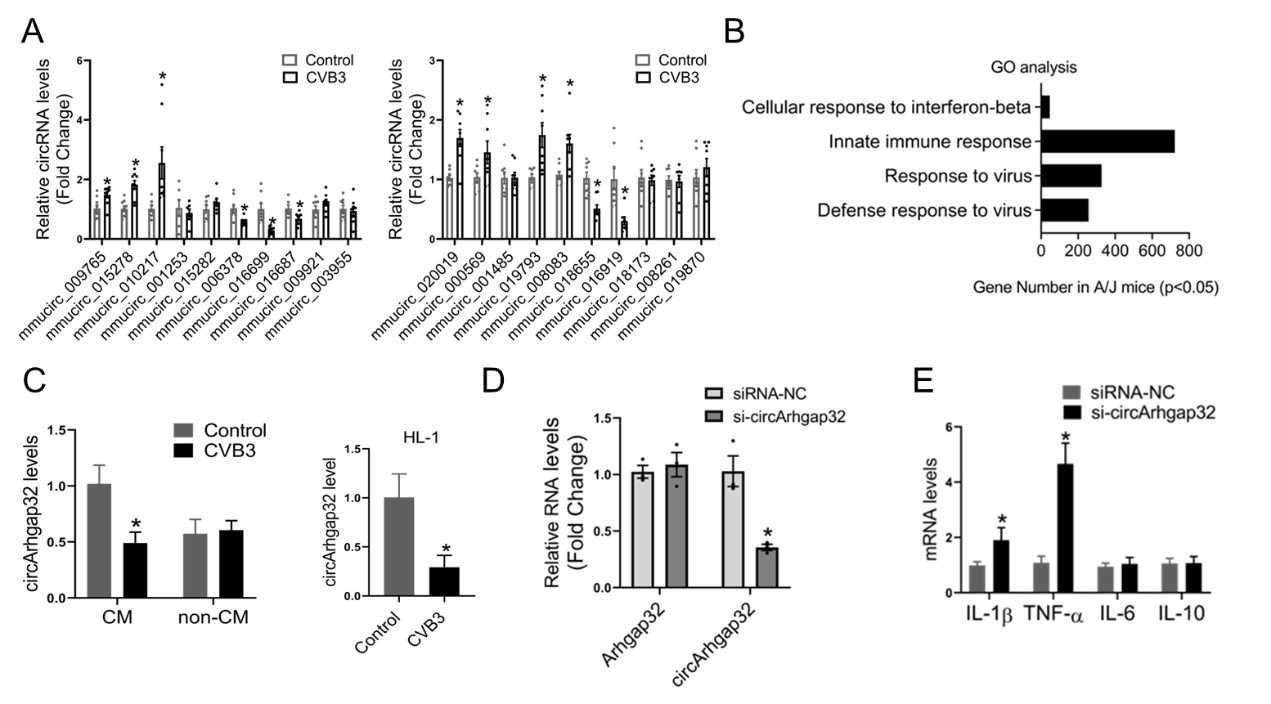


**Supplemental Figure 1.** (A) The expression levels of circRNAs were detected by qRT-PCR in HL-1 cells upon CVB3 infection; *P<0.05 VS. Control. (B) GO analysis for source genes of circRNAs in A/J mice. (C) The expression of circArhgap32 was detected by qRT-PCR; *P<0.05 VS. Control. (D) The expression of Arhgap32 and circArhgap32 were detected by qRT-PCR; *P<0.05 VS. siRNA-NC. (E) The expression levels of inflammatory factors were detected by qRT-PCR; *P<0.05 VS. siRNA-NC.
